# Vaccine effectiveness against laboratory-confirmed influenza hospitalizations among young children during the 2010-11 to 2013-14 influenza seasons in Ontario, Canada

**DOI:** 10.1371/journal.pone.0187834

**Published:** 2017-11-17

**Authors:** Sarah A. Buchan, Hannah Chung, Michael A. Campitelli, Natasha S. Crowcroft, Jonathan B. Gubbay, Timothy Karnauchow, Kevin Katz, Allison J. McGeer, J. Dayre McNally, David Richardson, Susan E. Richardson, Laura C. Rosella, Andrew Simor, Marek Smieja, Dat Tran, George Zahariadis, Jeffrey C. Kwong

**Affiliations:** 1 Dalla Lana School of Public Health, University of Toronto, Toronto, Ontario, Canada; 2 Institute for Clinical Evaluative Sciences, Toronto, Ontario, Canada; 3 Public Health Ontario, Toronto, Ontario, Canada; 4 Department of Laboratory Medicine and Pathobiology, University of Toronto, Toronto, Ontario, Canada; 5 The Hospital for Sick Children, Toronto, Ontario, Canada; 6 Children’s Hospital of Eastern Ontario, Ottawa, Ontario, Canada; 7 Department of Pathology and Laboratory Medicine, University of Ottawa, Ottawa, Ontario, Canada; 8 North York General Hospital, Toronto, Ontario, Canada; 9 Sinai Health System, Toronto, Ontario, Canada; 10 William Osler Health System, Brampton, Ontario, Canada; 11 Sunnybrook Health Sciences Centre, Toronto, Ontario, Canada; 12 McMaster University, Hamilton, Ontario, Canada; 13 Department of Paediatrics, University of Toronto, Toronto, Ontario, Canada; 14 London Health Sciences Centre, London, Ontario, Canada; 15 Newfoundland & Labrador Public Health Laboratory, St. John’s, Newfoundland & Labrador, Canada; 16 Department of Family & Community Medicine, University of Toronto, Toronto, Ontario, Canada; 17 University Health Network, Toronto, Ontario, Canada; University of Washington, UNITED STATES

## Abstract

Uncertainty remains regarding the magnitude of effectiveness of influenza vaccines for preventing serious outcomes, especially among young children. We estimated vaccine effectiveness (VE) against laboratory-confirmed influenza hospitalizations among children aged 6–59 months. We used the test-negative design in hospitalized children in Ontario, Canada during the 2010–11 to 2013–14 influenza seasons. We used logistic regression models adjusted for age, season, and time within season to calculate VE estimates by vaccination status (full vs. partial), age group, and influenza season. We also assessed VE incorporating prior history of influenza vaccination. We included specimens from 9,982 patient hospitalization episodes over four seasons, with 12.8% testing positive for influenza. We observed variation in VE by vaccination status, age group, and influenza season. For the four seasons combined, VE was 60% (95%CI, 44%-72%) for full vaccination and 39% (95%CI, 17%-56%) for partial vaccination. VE for full vaccination was 67% (95%CI, 48%-79%) for children aged 24–59 months, 48% (95%CI, 12%-69%) for children aged 6–23 months, 77% (95%CI, 47%-90%) for 2010–11, 59% (95%CI, 13%-81%) for 2011–12, 33% (95%CI, –18% to 62%) for 2012–13, and 72% (95%CI, 42%-86%) for 2013–14. VE in children aged 24–59 months appeared similar between those vaccinated in both the current and previous seasons and those vaccinated in the current season only, with the exception of 2012–13, when VE was lower for those vaccinated in the current season only. Influenza vaccination is effective in preventing pediatric laboratory-confirmed influenza hospitalizations during most seasons.

## Introduction

Rates of influenza-attributable hospitalizations are as high among young children as older adults [1]. Canada’s National Advisory Committee on Immunization (NACI) identifies children aged 6–23 months as a priority group for vaccination, and added children aged 24–59 months to the list of recommended recipients starting in the 2012–13 influenza season [2]. Uncertainty remains regarding vaccine effectiveness (VE) against serious outcomes, including hospitalizations and deaths, especially among young children. Indeed, the latest Cochrane review reported no conclusive evidence that influenza vaccines reduce hospitalizations among children younger than two years of age [3].

In addition, the impact of repeated vaccination on VE has been a topic of recent controversy,[4] but few studies have focused on children or hospitalized patients. Ohmit et al. found no negative impact of repeated vaccination on VE in children younger than 9 years of age in 2013–14 when A/H1N1 circulated. This contrasted with their results from the prior season when A/H3N2 dominated and VE was higher for those vaccinated in the current season only [5]. Thompson et al. examined this phenomenon in children younger than 8 years of age in 2012–13 and found similar VE estimates for those vaccinated in the current season only and those vaccinated in both the prior and current seasons [6].

The objective of this study was to evaluate influenza VE against hospitalizations for young children aged 6–59 months for the 2010–11 to 2013–14 seasons in Ontario, Canada. We also assessed the impact of repeated influenza vaccination in young children.

## Patients and methods

### Study population, setting, and design

We studied children aged 6–59 months who were hospitalized during the 2010–11 to 2013–14 influenza seasons in Ontario. Individual-level laboratory data were collected from a network of laboratories and linked using unique encoded identifiers to health administrative data, including hospital records and physician billing claims, at the Institute for Clinical Evaluative Sciences (ICES).

We estimated VE using the test-negative design (TND) [7, 8]. We included all respiratory specimens tested for influenza; those with laboratory-confirmed influenza served as cases and those testing negative served as controls. The study was restricted to periods when influenza was in circulation, based on a threshold level of 5% positivity on tested respiratory specimens for the province (S1 Table).

Ethics approval for this study was obtained from the Sunnybrook Health Sciences Centre Research Ethics Board and the Office of Research Ethics at the University of Toronto, Toronto, Canada. Written or verbal informed consent was not required from the subjects in this study, as ICES is permitted to obtain and use personal health information without patient consent for specific purposes, as outlined in Ontario’s *Personal Health Information Protection Act*. All data were fully anonymized before access by the researchers, and the requirement for informed consent was waived by the aforementioned ethics committees.

### Data sources and definitions

#### Laboratory testing

We included respiratory specimens tested at five hospitals and 11 public health laboratories distributed across the province. We included specimens tested using monoplex and multiplex polymerase chain reaction (PCR), viral culture, direct immunofluorescence assay (DFA), or enzyme immunoassay tests (EIA). In this dataset, 62.3% of children were tested using PCR, 22.0% with viral culture, 15.8% with DFA and 0.3% with EIA.

#### Hospitalizations

Hospitalizations were identified using the Canadian Institute of Health Information Discharge Abstract Database. We restricted the analysis to individuals with specimens collected within three days of hospital admission (96% of the sample) to minimize the inclusion of hospital-acquired influenza infections. We included one hospitalization with a specimen collected per individual per season. For individuals with multiple specimens collected during multiple hospitalizations within a season, we included the first hospitalization with a specimen positive for influenza (cases), or the first hospitalization if all specimens collected within the season were negative (controls).

#### Influenza vaccination

We used physician billing claims recorded in the Ontario Health Insurance Plan (OHIP) database to ascertain influenza vaccination status. Children were classified as fully vaccinated if they had two influenza vaccination billing claims in the current season with administration dates ≥28 days apart, or one billing claim in the current season with another billing claim in any prior season, and if the administration date of the dose(s) in the current season was ≥14 days before specimen collection date. Children were classified as partially vaccinated if they received two doses in the current season with the second <28 days after the first or <14 days before specimen collection date, or if they received only one of two recommended doses in the current season (≥14 days before specimen collection date). Both trivalent (TIV) and quadrivalent (QIV) inactivated vaccines are recommended for those aged 6–23 months but only TIV products were available in Ontario during the study period. For the 2011–12 to 2013–14 seasons, live attenuated inactivated vaccines (LAIV) were preferentially recommended over inactivated vaccines for healthy children aged 24–59 months, but they were not part of the publicly funded immunization program and therefore had minimal uptake [2].

#### Covariates

We used health administrative data to identify demographic characteristics, underlying health conditions (including asthma, diabetes, and cancer), and prior healthcare use, including past hospitalizations and continuity of care (defined as the percentage of primary care visits assigned to the child’s primary care provider). We used postal code of residence to assign neighbourhood income quintile (based on census-derived neighbourhood income) and rurality. We modified Feudtner et al.’s methodology [9] to suit the available data to assign complex chronic condition (CCC) status using physician billing and hospital claims (S2 Table). Birthweight and gestational age were determined from the mother’s delivery hospitalization record.

### Statistical analysis

We used logistic regression to estimate VE by comparing the odds of vaccination in the cases to the odds of vaccination in the controls. VE was calculated as (1–OR_adjusted_)x100%. We estimated VE for full and partial vaccination status separately. *A priori*, we controlled for age (in months), season, and time within season (month relative to peak) in the adjusted estimates [10, 11]. All potential confounders were evaluated for inclusion in the adjusted model using the Hosmer and Lemeshow forward model building strategy [12], but no additional covariates were included.

We tested for a difference in VE between fully and partially vaccinated children by calculating the odds ratio with those partially vaccinated as our reference [6]. We performed subgroup analyses by season, subtype, age group, sex, presence of a CCC, and time within season. In sensitivity analyses, we restricted the analysis to individuals with specimens collected during a hospitalization with an acute respiratory illness (ARI) diagnostic code recorded in any of the diagnosis fields in the hospitalization record (S3 Table); we evaluated the addition of any comorbidity (any CCC, diabetes, asthma, and/or cancer) on VE estimates; and we accounted for the possibility of misclassification of our exposure measurement [13], since not all influenza vaccinations are recorded in the OHIP database. As there are no published values for the sensitivity and specificity of physician billing codes for influenza vaccination of children younger than 5 years of age, we extrapolated measures from available data to this age group [14, 15]. To demonstrate specificity of the association between influenza vaccination and influenza hospitalization, we evaluated the association between influenza vaccination and laboratory-confirmation of one of four non-influenza respiratory viruses (respiratory syncytial virus, adenovirus, parainfluenza, or human metapneumovirus), for which no association was expected.

To assess the impact of repeated vaccination, we estimated VE for any vaccination in those aged 24–59 months by vaccination history using a categorical indicator variable for those vaccinated in both the current and prior seasons, the current season only, the prior season only, and neither season (i.e., four mutually exclusive groups).

We tested two main assumptions of the TND: 1) influenza testing is not associated with vaccination status; and 2) vaccination status is not associated with non-influenza respiratory viruses [7, 8, 16].

All analyses were conducted using SAS Enterprise Guide 6.1 (SAS Institute Inc., Cary, NC). All tests were two-sided and used p<0.05 as the level of statistical significance.

## Results

We included 9,982 hospitalization events during which a respiratory specimen was collected and tested for influenza for 9,547 unique children. A minority of children (4.1%) were included in more than one season. A total of 1,280 (12.8%) individuals had specimens that tested positive for influenza (range across seasons: 11.5%-14.5%). Across seasons, 1,151 (11.5%) children were classified as having received at least one influenza vaccination (range across seasons: 10.0%-13.8%), with 6.0% fully vaccinated and 5.6% partially vaccinated. Of the 882 individuals who tested positive for influenza A, 451 were subtyped, with 163 positive for A/H1N1 only, and 286 positive for A/H3N2 only. There were 402 individuals who tested positive for influenza B (including ≤5 influenza A/B coinfections); lineage information was not available. Influenza-positive children were more likely to be unvaccinated, older, reside in urban areas, and to be admitted during the peak influenza month (Table 1 and S4 Table). Children who had a CCC or asthma, a past intensive care unit (ICU) admission, or a history of low birthweight or preterm birth were more likely to be vaccinated (Table 2 and S5 Table).

**Table 1:** Characteristics of influenza test-positive and influenza test-negative hospitalized children.

Characteristic	Test-positive patients (n = 1,280)	Test-negative patients (n = 8,702)	p-value
Vaccination status			<.001
Fully vaccinated	36 (2.8%)	559 (6.4%)	
Partially vaccinated	45 (3.5%)	511 (5.9%)	
Unvaccinated	1,199 (93.7%)	7,632 (87.7%)	
Influenza season			<.001
2010–11	369 (28.8%)	2,168 (24.9%)	
2011–12	223 (17.4%)	1,330 (15.3%)	
2012–13	359 (28.0%)	2,681 (30.8%)	
2013–14	329 (25.7%)	2,523 (29.0%)	
Age (months), Median (IQR)	25 (15–41)	19 (12–31)	<.001
Age group			<.001
6–23 months	618 (48.3%)	5,421 (62.3%)	
24–59 months	662 (51.7%)	3,281 (37.7%)	
Male sex	754 (58.9%)	4,971 (57.1%)	0.23
Rural residence	85 (6.6%)	858 (9.9%)	<.001
Neighbourhood income quintile			0.25
1 (lowest)	321 (25.1%)	2,047 (23.5%)	
2	281 (22.0%)	1,777 (20.4%)	
3	253 (19.8%)	1,741 (20.0%)	
4	252 (19.7%)	1,737 (20.0%)	
5 (highest)	170 (13.3%)	1,331 (15.3%)	
No. of outpatient visits in past year, Mean ± SD	10.70 ± 8.45	10.83 ± 8.88	0.61
No. of hospitalizations in past year, Mean ± SD	0.83 ± 1.78	0.78 ± 1.43	0.28
Continuity of care, Mean ± SD	59.01 ± 27.89	62.86 ± 27.87	<.001
Risk factors for influenza complications			
Any complex chronic condition	344 (26.9%)	2,245 (25.8%)	0.41
Cancer	33 (2.6%)	188 (2.2%)	0.34
Diabetes	≤5 (≤1.0%)	32 (0.4%)	0.61
Asthma	392 (30.6%)	3,280 (37.7%)	<.001
Preterm birth	185 (14.5%)	1,452 (16.7%)	0.02
Low birthweight	179 (14.0%)	1,253 (14.4%)	0.22
Month of influenza test^a^			<.001
2 months before	≤5 (≤0.4%)^b^	48 (0.6%)	
1 month before	90 (7.0%)	1,037 (11.9%)	
Peak month	413 (32.3%)	1,832 (21.1%)	
1 month after	420 (32.8%)	1,701 (19.5%)	
2 months after	152 (11.9%)	1,680 (19.3%)	
3 months after	130 (10.2%)	1,324 (15.2%)	
4 months after	60 (4.7%)	763 (8.8%)	
5 months after	≤15 (≤1.2%)	317 (3.6%)	

^a^Time relative to peak month of influenza circulation.

^b^Some cells suppressed because of small cell size (direct or by inference), which cannot be reported as per privacy regulations.

IQR, interquartile range; SD, standard deviation

**Table 2:** Characteristics of fully vaccinated, partially vaccinated, and unvaccinated hospitalized children.

Characteristic	Fully vaccinated (n = 595)	Partially vaccinated (n = 556)	Unvaccinated (n = 8,831)	p-value
Influenza season				<.001
2010–11	122 (20.5%)	131 (23.6%)	2,284 (25.9%)	
2011–12	102 (17.1%)	87 (15.6%)	1,364 (15.4%)	
2012–13	169 (28.4%)	147 (26.4%)	2,724 (30.8%)	
2013–14	202 (33.9%)	191 (34.4%)	2,459 (27.8%)	
Age (months), Median (IQR)	25 (16–40)	17 (12–26)	20 (12–33)	<.001
Age group				<.001
6–23 months	279 (46.9%)	402 (72.3%)	5,358 (60.7%)	
24–59 months	316 (53.1%)	154 (27.7%)	3,473 (39.3%)	
Male sex	345 (58.0%)	319 (57.4%)	5,061 (57.3%)	0.95
Rural residence	30 (5.0%)	38 (6.8%)	875 (9.9%)	<.001
Neighbourhood income quintile				
1 (lowest)	118 (19.8%)	110 (19.8%)	2,140 (24.2%)	<.001
2	105 (17.6%)	113 (20.3%)	1,840 (20.8%)	
3	126 (21.2%)	105 (18.9%)	1,763 (20.0%)	
4	132 (22.2%)	116 (20.9%)	1,741 (19.7%)	
5 (highest)	114 (19.2%)	111 (20.0%)	1,276 (14.4%)	
No. of outpatient visits in past year, Mean ± SD	14.44 ± 9.47	15.32 ± 11.25	10.29 ± 8.47	<.001
No. of hospitalizations in past year, Mean ± SD	0.88 ± 1.59	1.06 ± 1.98	0.76 ± 1.43	<.001
Continuity of care, Mean ± SD	61.34 ± 26.80	60.80 ± 27.28	62.53 ± 28.01	0.24
Risk factors for influenza complications				
Any complex chronic condition	247 (41.5%)	193 (34.7%)	2,149 (24.3%)	<.001
Cancer	9 (1.5%)	13 (2.3%)	199 (2.3%)	0.48
Diabetes	6 (1.0%)	≤5 (≤1.0%)	28 (0.3%)	0.03
Asthma	268 (45.0%)	223 (40.1%)	3,181 (36.0%)	<.001
Preterm birth	140 (23.5%)	112 (20.1%)	1,385 (15.7%)	<.001
Low birthweight	135 (22.7%)	102 (18.3%)	1,195 (13.5%)	<.001
Month of influenza test^a^				<.001
2 months before	≤5 (≤0.8%)^b^	≤5 (≤0.9%)	43 (0.5%)	
1 month before	51 (8.6%)	54 (9.7%)	1,022 (11.6%)	
Peak	99 (16.6%)	133 (23.9%)	2,013 (22.8%)	
1 month after	109 (18.3%)	112 (20.1%)	1,900 (21.5%)	
2 months after	132 (22.2%)	114 (20.5%)	1,586 (18.0%)	
3 months after	106 (17.8%)	79 (14.2%)	1,269 (14.4%)	
4 months after	69 (11.6%)	41 (7.4%)	713 (8.1%)	
5 months after	≤30 (≤5.0%)	≤20 (≤3.6%)	285 (3.2%)	

^a^Time relative to peak month of influenza circulation.

^b^Some cells suppressed because of small cell size (direct or by inference), which cannot be reported as per privacy regulations.

IQR, interquartile range; SD, standard deviation.

The overall adjusted VE against laboratory-confirmed influenza hospitalization was 60.4% (95%CI, 44.0%-72.1%) for fully vaccinated children and 39.2% (95%CI, 16.6%-55.6%) for partially vaccinated children (Table 3; unadjusted results in S6 Table). Full vaccination offered statistically significant protection for three of the four influenza seasons studied, whereas partial vaccination offered protection for two seasons. VE for any vaccination was higher for children aged 24–59 months relative to those aged 6–23 months (p-value for interaction = 0.012). VE appeared higher for fully vaccinated children in all sub-analyses except against A/H3N2; however, the only statistically significant difference in VE by vaccination status was observed against influenza B (p = 0.03). Partial vaccination was not significantly protective against influenza for the 2011–12 or 2012–13 seasons, against A/H1N1 or influenza B, for children aged 6–23 months, or for children with asthma.

**Table 3:** Estimates of vaccine effectiveness by selected characteristics.

Analysis (cases/total)	Fully vaccinated	Partially vaccinated	Any vaccination
Overall (1280/9982)	60.4 (44.0, 72.1)	39.2 (16.6, 55.6)	50.8 (37.6, 61.2)
*Season*			
2010–11 (369/2537)	77.2 (46.9, 90.2)	69.1 (32.5, 85.9)	73.4 (52.5, 85.1)
2011–12 (223/1553)	59.0 (12.8, 80.8)	45.3 (−22.5, 75.5)	53.5 (18.5, 73.4)
2012–13 (359/3040)	33.1 (−18.4, 62.2)	−16.6 (−95.7, 30.6)	11.6 (−30.7, 40.3)
2013–14 (329/2852)	71.9 (42.1, 86.4)	47.0 (5.2, 70.4)	60.3 (37.1, 74.9)
*By influenza type/subtype*			
Influenza A (882/9584)	60.7 (38.9, 74.7)	50.5 (26.0, 66.9)	55.6 (40.0, 76.2)
A/H1N1 (164/8866)	82.1 (27.3, 95.6)	31.5 (−41.4, 66.8)	56.2 (16.3, 77.1)
A/H3N2 (287/8989)	53.3 (3.5, 77.4)	69.6 (25.2, 87.7)	61.3 (31.6, 78.1)
Influenza B (402/9104)^a^	58.0 (28.3, 75.4)	11.8 (−44.8, 46.2)	40.8 (14.3, 59.2)
*By age group*			
6–23 months (618/6039)	47.6 (11.9, 68.8)	27.6 (−5.0, 50.0)	35.6 (12.4, 52.6)
24–59 months (662/3943)	67.1 (47.5, 79.4)	58.8 (24.7, 77.5)	64.4 (48.2, 75.5)
*By sex*			
Females (526/4257)	63.8 (35.7, 79.6)	49.3 (13.1, 70.4)	57.2 (36.2, 71.2)
Males (754/5725)	58.9 (36.2, 73.5)	32.8 (0.5, 54.6)	47.1 (28.8, 60.7)
*By complex chronic condition*			
Yes (344/2589)	62.9 (36.2, 78.4)	45.1 (6.5, 67.8)	55.4 (34.2, 69.8)
No (936/7393)	57.7 (33.3, 73.1)	36.8 (6.4, 57.4)	47.6 (29.1, 61.2)
*By asthma*			
Yes (392/3672)	57.1 (26.1, 75.1)	23.0 (−28.0, 53.7)	43.3 (17.1, 61.2)
No (888/6310)	60.9 (38.4, 75.2)	45.5 (18.2, 63.7)	53.5 (36.7, 65.8)
*Peak month*			
Yes (413/2245)	68.4 (33.8, 84.9)	44.4 (1.6, 68.6)	56.2 (30.8, 72.3)
No (867/7737)	59.8 (40.6, 72.8)	36.8 (8.1, 56.6)	50.2 (34.4, 62.6)
*Sensitivity analyses*			
Restricted to ARI-coded hospitalizations (1183/8760)	59.9 (42.6, 72.0)	34.4 (9.1, 52.6)	48.6 (34.4, 59.8)
Included term for any comorbidity in model (1280/9982)	58.6 (41.4, 70.8)	37.0 (13.6, 54.1)	48.8 (35.0, 59.6)
Other respiratory virus positive (1600/3927)^b^	15.2 (−12.1, 35.9)	16.2 (−10.5, 36.5)	15.8 (−3.2, 31.2)

^a^n = 24 specimens were not tested for influenza B.

^b^Restricted to those who were tested for respiratory syncytial virus, parainfluenza virus, adenovirus, and human metapneumovirus.

VE estimates were unchanged when we restricted to children with specimens collected during an ARI-coded hospitalization and when we included any comorbidity in our model (Table 3). When we corrected for misclassification of our exposure measurement based on the most plausible estimates of sensitivity (77%) and specificity (98%), VE for any vaccination changed from 50.8% (95%CI, 37.6%-61.2%) to 60.7% (95%CI, 47.9%-70.6%). We performed these sensitivity analyses on a range of estimates to determine their potential impact on VE (S1 Fig). Influenza vaccination was not associated with hospitalization for non-influenza respiratory viruses.

**Fig 1:**
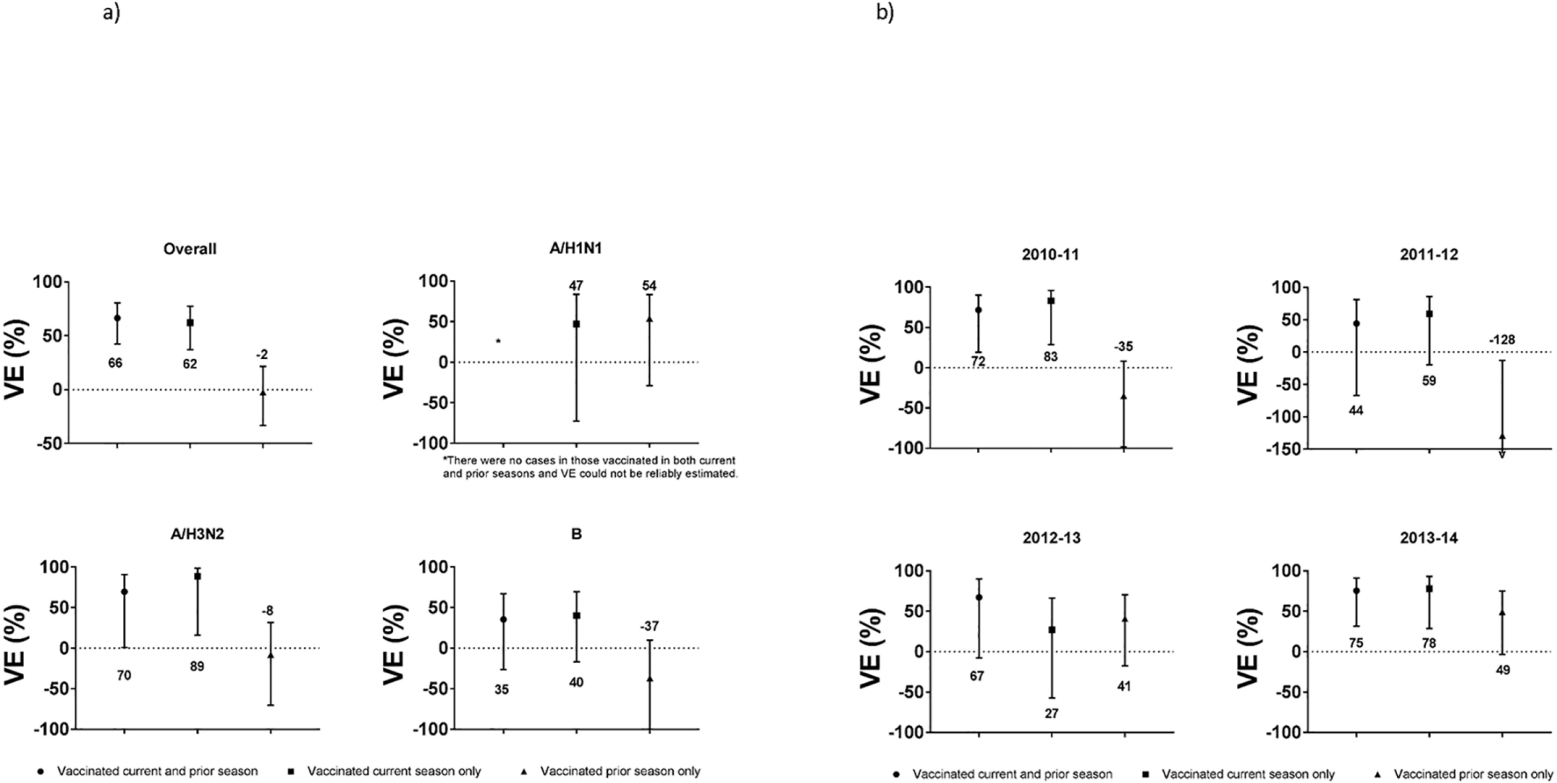
Adjusted VE for any vaccination in children aged 24–59 months, by vaccination status during the current and/or the prior influenza seasons, a) by influenza subtype and b) by influenza season. Vaccine effectiveness estimates are presented for children vaccinated in the current and prior season (circles), the current season only (squares), and the prior season only (triangles).

When examining the influence of prior vaccinations status in children aged 24–59 months, VE estimates for those vaccinated in the current season only versus two sequential seasons were similar (Fig 1a), though there was variation by year (Fig 1b). In the 2010–11 and 2011–12 seasons and for A/H3N2, VE appeared higher for those vaccinated in the current season only, though the reverse was seen in the 2012–13 season; however, confidence intervals were wide due to small numbers. Confidence intervals were also wide when examining residual protection based on prior season vaccination only.

For patients who had an ARI code associated with their hospitalization, 46% were tested for influenza. Among these ARI-coded hospitalizations, 12.6% of patients who were tested for influenza were vaccinated, compared to 12.4% of patients who were not tested for influenza (p = 0.56), confirming the absence of an association between influenza testing and vaccination status. Using the 95% of our sample that were tested for at least one other respiratory virus, 32.2% of unvaccinated children tested positive for another respiratory virus compared to 32.1% of children with any vaccination (p = 0.96), confirming the absence of an association between vaccination status and non-influenza respiratory viruses. This assumption held for fully, partially, and unvaccinated children, as well as between age groups and across seasons.

## Discussion

During the 2010–11 to 2013–14 influenza seasons, vaccination reduced the risk of laboratory-confirmed influenza hospitalizations by 60% for fully vaccinated children aged 6–59 months and by 39% for partially vaccinated children in Ontario. We observed statistically significant VE for fully vaccinated children for all seasons except 2012–13, and for all subgroups except those infected by A/H3N2. We did not detect statistically significant VE for partially vaccinated children during the 2011–12 and 2012–13 seasons, or for those aged 6–23 months, those with asthma, and those with influenza A/H1N1 or B infections.

Our estimates are generally consistent with previous studies that have assessed VE among young children for these four seasons (Table 4), but direct comparisons are difficult due to varying healthcare settings (inpatient versus outpatient), geographical locations, and age groups studied [5, 17–35]. The sole previous study of VE against laboratory-confirmed influenza hospitalizations specifically in young children aged 6–59 months reported an estimate of 75% (95%CI, –100% to 97%) for the 2012 southern hemisphere influenza season in New Zealand [33]. Our estimate for 2011–12 of 54% (95%CI, 19%-73%) is well within the wide confidence interval of that study. Assuming that VE estimates for hospitalization and outpatient outcomes should be consistent, as suggested in a recent systematic review [36], our results are remarkably similar to estimates from the United Kingdom for 2010–11 (VE = 73%; 95%CI, 53%-85% versus 72%; 95%CI, 12%-91%) and 2011–12 (VE = 54%; 95%CI, 19%-73% versus 52%; 95%CI, –446% to 96%), but our estimates had tighter confidence intervals [22, 27]. Relaxing the age criteria to include children as old as 19 years, our estimates are congruent with estimates from North America, Europe, Asia, and Australasia for the 2010–11, 2011–12, and 2013–14 seasons [5, 17–20, 25, 29, 31, 34, 35].

**Table 4:** Comparison with other published vaccine effectiveness estimates, by season, and other study characteristics.

Influenza season	Author, Year	Setting	Location	Age group	Adjusted VE (95% CI)	Ontario VE
**2010–11**	Cowling, 2014 [19]	Inpatient	Hong Kong	6mo-17 years	84 (44, 96)	**73 (53, 85)**
	Chung, 2016 [18]	Outpatient	USA (Flu VE Network)	2–8 years	70 (53, 81)	
	Kafatos, 2013 [22]	Outpatient	United Kingdom	<5 years	72 (12, 91)	
	Englund, 2013 [20]	Outpatient	Germany	0–14 years	84 (24, 97)	
**2011–12**	Turner, 2014 [33]	Inpatient	New Zealand	6mo-5 years	75 (–100, 97)	**54 (19, 73)**
	Cowling, 2014 [19]	Inpatient	Hong Kong	6mo-17 years	51 (10, 74)	
	Menniti-Ipolito, 2014 [25]	ED/Inpatient	Italy	6mo-16 years	41 (–126, 84)	
	Chung, 2016 [18]	Outpatient	USA (Flu VE Network)	2–8 years	51 (22, 69)	
	Skowronski, 2014 [31]	Outpatient	Canada	1–19 years	64 (23, 84)	
	Pebody, 2013 [27]	Outpatient	United Kingdom	<5 years	52 (–446, 96)	
	Kissling, 2013 [24]	Outpatient	Europe (I-MOVE)	<15 years	19 (–170, 76)	
	Wang, 2016 [35]	Outpatient/ED	China	6–59 months	67 (41, 82)	
**2012–13**	Turner, 2014 [32]	Inpatient	New Zealand	6mo-17 years	78 (2, 95)	**12 (–31, 40)**
	Cowling, 2014 [19]	Inpatient	Hong Kong	6mo-17 years	81 (37, 94)	
	Menniti-Ipolito, 2014 [25]	ED/Inpatient	Italy	6mo-16 years	26 (–153, 78)	
	Chung, 2016 [18]	Outpatient	USA (Flu VE Network)	2–8 years	46 (27, 60)	
	Kissling, 2014 [23]	Outpatient	Europe (I-MOVE)	0–14 years	36 (–41, 71)*	
	Fu, 2015 [21]	Outpatient	China	8mo-6 years	67 (58, 74)^†^	
	Skowronski [30]	Outpatient	Canada	1–19 years	87 (65, 95)	
	Ohmit, 2015[26]	Outpatient	USA (HIVE)	<9 years	–4 (–110, 49)	
**2013–14**	Blyth, 2016 [17]	Inpatient	Australia	6mo-16 years	56 (12, 78)	**60 (37, 75)**
	Pierse, 2016 [28]	Inpatient	New Zealand	6mo-17 years	–30 (–212, 46)	
	Chung, 2016 [18]	Outpatient	USA (Flu VE Network)	2–8 years	61 (34, 77)	
	Skowronski, 2015 [29]	Outpatient	Canada	1–19 years	77 (47, 90)	
	Valenciano, 2015 [34]	Outpatient	Europe (I-MOVE)	0–14 years	64 (–86, 93)	
	Ohmit, 2016 [5]	Outpatient	USA (HIVE)	<9 years	68 (10, 88)^†^	

*Estimate is for H3N2 only;

^†^Estimate is for H1N1 only.

ED, emergency department

In contrast, our estimate for the 2012–13 season was consistent with some estimates (that may have included outpatients and/or older children) from USA and Europe [23, 25, 26], but not estimates from Canada, China, Hong Kong, or New Zealand [18, 19, 21, 30, 32]. In Canada, mutations in the egg-adapted A/H3N2 vaccine strain resulted in lowered VE [30]. The Influenza-Monitoring Vaccine Effectiveness (I-MOVE) network found low VE in 2012–13 for children aged <14 years who received inactivated split vaccine [23]. Since most influenza vaccines distributed in Canada are also split vaccines, this could be a potential explanation, although it is still inconsistent with another Canadian estimate, albeit one that included older children and data from other provinces [30].

Given the interest in examining whether influenza vaccination impacts severity of infection, we compared the estimates from our inpatient study to other Canadian outpatient estimates (for children aged 1–19 years) [29–31]. Our estimates were similar with overlapping confidence intervals for 2011–12 (VE = 64%; 95%CI, 23%-84% in outpatients versus 54%; 95%CI, 19%-73% in inpatients) and 2013–14 (VE = 77%; 95%CI, 47%-90% in outpatients versus 60%; 95%CI, 37%-75% in inpatients), but not for 2012–13 (VE = 87%; 95%CI, 65%-95% in outpatients versus 12%; 95%CI, –31%-40% in inpatients). Understanding such discrepancies deserves further study.

We found higher VE against A/H3N2 in partially vaccinated children relative to those fully vaccinated, which is consistent with previous work [6, 37]. This may be because children are defined as fully vaccinated either through receipt of two doses in their first season or one dose if they had been vaccinated in any prior season. Differentiating the effect of partial vaccination versus repeated vaccination is challenging in this age group as we may be inherently studying the effect of repeated vaccination when examining fully vaccinated children. For A/H3N2, we estimated higher VE for those vaccinated in the current season only, which is equivalent to partial vaccination in certain circumstances. In the future, studies focusing on this age group should differentiate between the two types of full vaccination when estimating VE.

Among children aged 24–59 months, we found no difference in VE between those vaccinated in the current season only compared to those vaccinated in both prior and current seasons. However, we were limited by our sample size from evaluating the impact of repeated vaccination by subtype. This analysis could also only consider vaccination in two consecutive seasons rather than serial vaccination, although the number of vaccines in this age group would be limited. Our results may not generalize to older individuals with longer histories of both influenza and influenza vaccine exposure.

Our study has several strengths. We were able to test some of the assumptions that are routinely mentioned in test-negative studies but never evaluated. Specifically, we confirmed that vaccination status was not associated with influenza testing and that influenza vaccination was not associated with testing positive for other respiratory viruses. Pooling data across several years allowed for the evaluation of VE in selected subgroups of interest, and the nature of the vaccination data permitted evaluation of the impact of repeated vaccination in a population with limited previous vaccine exposure. The size of our study population was enhanced by including numerous types of high-specificity laboratory tests. While values for sensitivity and specificity may vary by testing method, in TND studies with laboratory-confirmed outcomes, specificity is more important in terms of bias [38].

This study had several limitations. Symptom onset date was only available for the minority (16%) of specimens, but since children shed higher levels of virus for longer periods of time and we restricted to those tested within three days of hospital admission, absence of symptom onset date is less likely to be an issue in this age group [39]. Further, there was no strict case definition for initiating influenza testing, though this would not be expected to be different between test-positives and test-negatives. Further, 88% of individuals with specimens tested were collected during an ARI-coded hospitalization, which may serve as a proxy for testing criteria. Our VE estimates were unchanged when restricting the sample to individuals with specimens collected during ARI-coded hospitalizations. Vaccination status was determined from physician billing claims; children who received a vaccination at public health clinics may have been misclassified. Children in this age group are not eligible for vaccination through pharmacists and the majority (77%) of children in this age group receive influenza vaccination through physicians [14]. We would not expect differential misclassification by influenza case status, and our VE estimates would therefore be conservative. While we may have underestimated coverage relative to other studies, our data reflect vaccination status relative to the date of influenza testing in a hospitalized cohort, and do not reflect estimates of vaccine coverage for the influenza season [14]. Further, the vaccinated proportion is similar to that of other Canadian studies [29–31], though differences in setting and vaccination data sources may explain any residual differences. Differentiating TIV, QIV, and LAIV was not possible with the available data. However, neither LAIV nor QIV were publicly funded in Ontario until the 2015–16 influenza season, therefore most children in this study would have received TIV. We were limited by low influenza vaccine coverage, which impacted our power to detect significant VE in certain groups, especially when considering the importance of evaluating VE by full and partial vaccination status [40]. Coverage appeared low in our sample relative to other estimates, perhaps because some vaccinations may have not been documented or because previous survey results over-estimated coverage [14]. Compared with surveillance-based methods, this approach is insufficiently timely to produce mid-season or end-of-season results to inform program planning. Last, the subtype-specific estimates were limited because only half of individuals positive for influenza A had specimens that were subtyped.

## Conclusion

Our study demonstrates VE against laboratory-confirmed influenza hospitalizations for children aged 6–59 months. Despite variation across subgroups, we observed substantial protection for vaccinated children, particularly if fully vaccinated. These results support current recommendations to promote vaccination in this high-risk group.

### Disclaimers

This study was supported by the Institute for Clinical Evaluative Sciences (ICES) and Public Health Ontario (PHO), which are funded by annual grants from the Ontario Ministry of Health and Long-Term Care (MOHLTC). The opinions, results, and conclusions reported in this paper are those of the authors and are independent from the funding sources. Parts of this material are based on data and information compiled and provided by the Canadian Institute of Health Information (CIHI) and by Cancer Care Ontario (CCO). However, the analyses, conclusions, opinions, and statement expressed herein are those of the authors, and not necessarily those of CIHI or CCO. No endorsement by ICES, PHO, MOHLTC, CIHI, or CCO is intended or should be inferred.

## Supporting information

S1 Table Influenza circulation periods, recommended vaccine strains, and dominant circulating subtypes, by season. (DOCX)

S2 Table Classification of complex chronic conditions derived from Feudtner’s and applied to CIHI-DAD at ICES, including codes applied to ICD-10-CA. (DOCX)

S3 Table List of included ICD-10-CA codes to define ARI hospitalizations. (DOCX)

S4 Table Additional descriptive characteristics of influenza test-positive and influenza test-negative hospitalized children. (DOCX)

S5 Table Additional descriptive characteristics of fully vaccinated, partially vaccinated and unvaccinated hospitalized children. (DOCX)

S6 Table Unadjusted vaccine effectiveness estimates. (DOCX)

S1 Fig Impact of misclassification of vaccination status on VE estimates, by a) changing sensitivity and b) changing specificity. (DOCX)
