# Supplementary material for: Vaccine effectiveness against laboratory-confirmed influenza hospitalizations among young children during the 2010-11 to 2013-14 influenza seasons in Ontario, Canada
Source: PLoS One. 2017 Nov 17;12(11):e0187834. doi: 10.1371/journal.pone.0187834 (PMC5693284; doi:10.1371/journal.pone.0187834)
Supplement: S1 Table — (DOCX) [file pone.0187834.s001.docx]

**S1 Table.** Influenza circulation periods, recommended vaccine strains, and dominant circulating subtypes, by season

| Influenza season | Influenza Circulation Period | Peak week | WHO recommended vaccine strains [1] | Dominant circulating subtype(s) in Ontario^a^ |
| --- | --- | --- | --- | --- |
| 2010-11 | November 14, 2010 to April 9, 2011 | December 26, 2010 | A/California/7/2009 (H1N1), A/Perth/16/2009 (H3N2) and B/Brisbane/60/2008 (Victoria) | A/H3N2 |
| 2011-12 | January 29, 2012 to  May 19, 2012 | March 11, 2012 | A/California/7/2009 (H1N1), A/Perth/16/2009 (H3N2) and B/Brisbane/60/2008 (Victoria) | Influenza B |
| 2012-13 | November 4, 2012 to  May 11, 2013 | December 23, 2012 | A/California/7/2009 (H1N1), A/Victoria/361/2011 (H3N2) and B/Wisconsin/1/2010 (Yamagata) | A/H3N2 |
| 2013-14 | December 1, 2013 to  May 24, 2014 | December 29, 2013 | A/California/7/2009 (H1N1), A/Texas/50/2012 (H3N2) and B/Massachusetts/2/2012 (Yamagata) | A/H1N1 & Influenza B |

^a^As determined through Public Health Ontario’s Respiratory Pathogen Bulletins [2]

References

1. World Health Organization. WHO recommendations on the composition of influenza virus vaccines. Available at: <http://www.who.int/influenza/vaccines/virus/recommendations/en/>. Accessed November 17, 2016.

2. Public Health Ontario. Respiratory Pathogen Bulletin. Available at: http://www.publichealthontario.ca/en/ServicesAndTools/SurveillanceServices/Pages/Ontario-Respiratory-Virus-Bulletin.aspx . Accessed February 1, 2017.
