# Supplementary material for: Vaccine effectiveness against laboratory-confirmed influenza hospitalizations among young children during the 2010-11 to 2013-14 influenza seasons in Ontario, Canada
Source: PLoS One. 2017 Nov 17;12(11):e0187834. doi: 10.1371/journal.pone.0187834 (PMC5693284; doi:10.1371/journal.pone.0187834)
Supplement: S2 Table — (DOCX) [file pone.0187834.s002.docx]

**S2a Table**. Classification of complex chronic conditions derived from Feudtner’s method[3] and applied to CIHI-DAD at ICES

| **Complex Chronic Condition** | **Source** | **Alterations** |
| --- | --- | --- |
| Neurologic/Neuromuscular | Feudtner 2014 |  |
| Cardiovascular | Feudtner 2014 |  |
| Respiratory | Feudtner 2014 |  |
| Urorenal | Feudtner 2014 |  |
| Gastrointestinal | Feudtner 2014 |  |
| Hematology and immunodeficiency | Feudtner 2014 |  |
| Metabolic | Feudtner 2014 |  |
| Premature/Neonatal | Feudtner 2014 |  |
| Other/NOS (i.e. transplants) | Feudtner 2014 |  |
| Other congenital or genetic defect | Feudtner 2014 | Also included ICD-10 Chapter XVII Congenital malformations, deformations and chromosomal abnormalities (Q00-Q99) |
| Technology Assistance | Cohen 2012[4] | Removed device codes from Feudtner and replaced with TA codes derived from Canadian Classification of Intervention (CCI) codes |
| Malignancy | Ontario Cancer Registry | Removed malignancy category from Feudtner and used Ontario Cancer Registry (OCR) instead |

**S2b Table**. Codes of complex chronic conditions using Feudtner’s [3] methodology and applied to ICD-10-CA codes

| CIHI-DAD was used to identify children with a chronic complex condition diagnostic code in any hospitalization record since birth.  Neurological/Neuromuscular  E750, E751, E752, E754, F71, F72, F73, F842, G111, G112, G114, G118, G119, G120, G121, G122, G128, G129, G3101, G3109, G318, G3189, G3289, G71, G72, G80, G901, G938, G939, G94, Q00, Q01, Q02, Q03, Q04, Q05, Q06, Q07, G911, G319, G253, G9519, G9589, G909, G40311, G40301, G40211, G40219, G40411, G40419, G40804, G40111, G40119, G40911, G40919, G371, G372, G378, G8190, G8290, G8250, G8251, G8252, G8253, G8254, G835, G839, G931, G935, I6330, I6350, G10, G20, G210, G2111, G2119, G218, G230, G231, G232, G238, G2402, G248, G253, G254, G255, G2581, G2582, G2583, G2589, G259, G803, R403, G9782, T8509XA, T85190A, T85192A, T85199A, T8579XA, Z982, Z4541, Z4542, G310, G328, G951, G958, G4031, G4030, G4021, G4041, G4080, G4011, G4091, G82, I633, I635, G211, G240, G258, R4020, G978, G113, G318, Q851  Cardiovascular  I270, I271, I272, I2781, I2789, I279, I340, I348, I360, I368, I370, I378, I42, I43, I44, I45, I47, I48, I490, I491, I493, I494, I495, I498, I499, I509, I515, I517, I5181, I63139, I63239, Q20, Q212, Q213, Q214, Q218, Q22, Q23, Q24, Q251, Q252, Q253, Q254, Q255, Q256, Q257, Q26, R001, Q282, Q283, Q289, Z951, T82519A, T82529A, T82539A, T82599A, T82110A, T82111A, T82120A, T82121A, T82190A, T82191A, T8201XA, T8202XA, T8203XA, T8209XA, T82211A, T82212A, T82213A, T82218A, T82221A, T82222A, T82223A, T82228A, T82518A, T82528A, T82538A, T82598A, T826XXA, T827XXA, Z941, Z950, Z952, Z95810, Z95811, Z95812, Z95818, Z953, Z45010, Z45018, Z4502, Z4509, Z959, I278, I518, I631, I632, T86200, T86201, T86202, T86300, T86301, T86302, Q219, Q258, Q259, Z943  Respiratory  E84, G4753, I2782, I43, Q30, Q31, Q32, Q33, Q34, P280, Z902, J84112, J9500, J9501, J9502, J9503, J9504, J9509, J9620, Z430, Z930, Z942, Z990, J95850, Z9911, Z9912, T86819, I278, J841, J9503, J96, J95, T86800, T86801, T86802  Uro-renal  N18, Q60, Q61, Q62, Q63, Q64, Z905, Z906, G834, N312, N319, T8571XA, Z940, Z9350, Z9351, Z9352, Z9359, Z936, Z9115, Z992, Z435, Z436, Z446, Z4901, Z4902, Z4931, Z4932, T8610, T8611, T8612, Z49, Z490  Gastrointestinal  K50, K51, K73, K74, K754, K760, K761, K762, K763, K768, Q390, Q391, Q392, Q393, Q394, Q41, Q42, Q43, Q44, Q45, I820, K551, K562, K593, Z980, Z903, Z9049, K9420, K9422, K9423, K9429, Z944, Z9482, Z9483, Z931, Z932, Z933, Z934, Z431, Z432, Z433, Z434, Z4651, Z4659, T8640, T8641, T8642, T86890, T86891, T86899, T86850, T86851, T86859, Z904, K765, T86810, T86811, T86812  Hematology and immunodeficiency  B20, D55, D56, D57, D58, D60, D61, D71, D720, D80, D81, D82, D83, D84, D85, D87, D88, D86, M303, M359, B21, B22, B23, B24, D700, D704, D66, D682, D6941, D6942, D761, D762, D763, D869, M300, M310, M311, M3130, M314, M316, M3210, M3390, M340, M341, M349, D694, M321, M339, D89, D694  Metabolic  D841, E700, E702, E703, E704, E705, E708, E710, E711, E712, E713, E714, E715, E720, E721, E722, E723, E724, E728, E729, E740, E741, E742, E743, E744, E748, E749, E75, E760, E761, E762, E763, E770, E771, E780, E781, E782, E783, E784, E785, E786, E787, E788, E789, E791, E798, E804, E805, E806, E807, E830, E831, E833, E834, E88, H498, E85, E009, E230, E232, E222, E233, E237, E240, E242, E243, E248, E249, E2681, E250, E258, E259, Z4681, Z794, Z9641, E70, E71, E78, E268  Premature/Neonatal  P0501, P0511, P0502, P0512, P052, P059, P0701, P0702, P0721, P0722, P0723, P0724, P0725, P100, P101, P104, P524, P528, P115, P84, P916, P210, P250, P251, P253, P258, P219, P270, P271, P278, P350, P351, P2521, P2522, P560, P570, P578, P613, P614, P773, P832, P912, P05, P070, P072, P21, P252, P77  Other/NOS (i.e., transplants)  T8509XA, T85190A, T85192A, T85199A, T8579XA, Z982, Z45441, Z45442, T82519A, T82529A, T82539A, T82599A, T82110A, T82111A, T82120A, T82121A, T82190A, T82191A, T8201XA, T8202XA, T8203XA, T8209XA, T82211A, T82212A, T82213A, T82218A, T82221A, T82222A, T82223A, T82228A, T82518A, T82528A, T82538A, T82598A, T826XXA, T827XXA, Z950, Z952, Z953, Z95810, Z95811, Z95812, Z95818, Z45010, Z45018, Z4502, Z4509, Z959, J9500, J9501, J9502, J9503, J9504, J9509, Z430, Z930, Z942, Z990, J95850, Z9911, Z9912, T8571XA, Z940, Z9350, Z9351, Z9352, Z9359, Z936, Z9115, Z992, Z435, Z436, Z446, K9420, K9422, K9423, K9429, Z931, Z932, Z933, Z934, Z431, Z432, Z433, Z434, Z4651, Z4659, Z4681, Z9641, T84019A, T84029A, T84039A, T84049A, T84059A, T84069A, T84099A, T84498A, T84119A, T84129A, T84199A, T84498A, T8450XA, T8460XA, T847XXA, T8690, T8691, T8692, T8699, T8610, T8611, T8612, T8640, T8641, T8642, T8620, T8621, T8622, T86810, T86811, T86819, T8600, T8601, T8602, T8609, T86890, T86891, T86899, T86850, T86851, T86859, T865, T86890, T86891, T86899, T870X9, T871X9, T872, Y831, Y833, T8600, T8601, T8602, T8609, T8610, T8611, T8612, T8620, T8621, T8622, T86810, T86811, T86819, T8640, T8641, T8642, T86890, T86891, T86899, T86850, T86851, T86859, T865, T8690, T8691, T8692, T8699, T86890, T86891, T86899, T868, T869, T860, T87099, T87100, Z941, Z943, Z944, Z9480, Z9481, Z9482, Z9483, Z9488, Z949  Other congenital or genetic defect  E343, K449, M410, M412, M4130, M418, M419, M4330, M965, Q722, Q750, Q752, Q759, Q760, Q761, Q762, Q764, Q765, Q766, Q767, Q77, Q780, Q781, Q782, Q783, Q784, Q788, Q789, Q790, Q791, Q792, Q793, Q794, Q799, Q795, Q8740, Q8781, Q8789, Q897, Q899, Q909, Q913, Q914, Q917, Q928, Q93, Q950, Q969, Q97, Q98, Q992, Q998, Q999, Q898, Q81, M433, Q874, Q878, Q795, Q871, Q872, Q873 |
| --- |

References

3. Feudtner C, Feinstein JA, Zhong WJ, Hall M, Dai DW. Pediatric complex chronic conditions classification system version 2: updated for ICD-10 and complex medical technology dependence and transplantation. BMC Pediatr **2014**; 14: 199.

4. Cohen E, Berry JG, Camacho X, Anderson G, Wodchis W, Guttmann A. Patterns and costs of health care use of children with medical complexity. Pediatrics **2012**; 130(6): e1463-70.
