# Supplementary material for: Vaccine effectiveness against laboratory-confirmed influenza hospitalizations among young children during the 2010-11 to 2013-14 influenza seasons in Ontario, Canada
Source: PLoS One. 2017 Nov 17;12(11):e0187834. doi: 10.1371/journal.pone.0187834 (PMC5693284; doi:10.1371/journal.pone.0187834)
Supplement: S3 Table — (DOCX) [file pone.0187834.s003.docx]

**Table S3.** List of included ICD-10-CA codes to define ARI hospitalizations

| **Description** | **ICD-10 Code** |
| --- | --- |
| Viral infection, unspecified site | B34 |
| Viral agents as the cause of diseases classified to other chapters | B97 |
| Acute nasopharyngitis (common cold) | J00 |
| Acute sinusitis | J01 |
| Acute pharyngitis | J02 |
| Acute tonsillitis | J03 |
| Acute laryngitis and tracheitis | J04 |
| Acute obstructive laryngitis (croup) and epiglottitis | J05 |
| Acute upper respiratory infections of multiple or unspecified sites | J06 |
| Influenza due to identified novel influenza A virus | J09 |
| Influenza | J10, J11 |
| Viral pneumonia | J12 |
| Bacterial pneumonia | J13, J14, J15 |
| Pneumonia due to other specified organism | J16 |
| Pneumonia in infectious diseases classified elsewhere | J17 |
| Bronchopneumonia, organism unspecified | J18 |
| Acute bronchitis and bronchiolitis | J20, J21 |
| Unspecified acute lower respiratory infection | J22 |
| Other diseases of upper respiratory tract | J39.8, J39.9 |
| Bronchitis, not specified as acute or chronic | J40 |
| Asthma | J45 |
| Adult respiratory distress syndrome | J80 |
| Pulmonary edema | J81 |
| Respiratory failure | J96 |
| Other respiratory disorders | J98 |
| Cough | R05 |
| Shortness of breath (dyspnea) | R06.0 |
| Stridor | R06.1 |
| Wheezing | R06.2 |
| Tachypnea | R06.4 |
| Sneezing | R06.7 |
| Respiratory abnormality, other | R06.8 |
| Pain in throat | R07.0 |
| Chest pain on breathing | R07.1 |
| Respiratory arrest | R09.2 |
| Abnormal sputum | R09.3 |
| Fever | R50 |
| Malaise and fatigue | R53 |
