# Supplementary material for: Vaccine effectiveness against laboratory-confirmed influenza hospitalizations among young children during the 2010-11 to 2013-14 influenza seasons in Ontario, Canada
Source: PLoS One. 2017 Nov 17;12(11):e0187834. doi: 10.1371/journal.pone.0187834 (PMC5693284; doi:10.1371/journal.pone.0187834)
Supplement: S4 Table — (DOCX) [file pone.0187834.s004.docx]

**S4 Table.** Additional characteristics of influenza test-positive and influenza test-negative hospitalized children

| **Characteristic** | **Test-positive patients (n=1,280)** | **Test-negative patients (n=8,702)** | **p-value** |
| --- | --- | --- | --- |
| Complex chronic conditions |  |  |  |
| Neurologic/Neuromuscular | 53 (4.1%) | 423 (4.9%) | 0.26 |
| Cardiovascular | 68 (5.3%) | 505 (5.8%) | 0.48 |
| Respiratory | 48 (3.8%) | 350 (4.0%) | 0.64 |
| Urorenal | 34 (2.7%) | 241 (2.8%) | 0.82 |
| Gastrointestinal | 54 (4.2%) | 458 (5.3%) | 0.11 |
| Hematologic/Immunodeficiency | 71 (5.5%) | 357 (4.1%) | 0.02 |
| Metabolic | 35 (2.7%) | 240 (2.8%) | 0.96 |
| Premature/Neonatal | 129 (10.1%) | 877 (10.1%) | 1.0 |
| Other Congenital | 213 (16.6%) | 1,518 (17.4%) | 0.48 |
| Other/Not specified | 60 (4.7%) | 471 (5.4%) | 0.28 |
| Technology Assistance | 61 (4.8%) | 503 (5.8%) | 0.14 |
|  |  |  |  |
| ICU admission | 73 (5.7%) | 554 (6.4%) | 0.36 |
|  |  |  |  |
| Past ICU admission | 129 (10.1%) | 1,011 (11.6%) | 0.11 |
| ICU, intensive care unit |  |  |  |
