# Supplementary material for: Vaccine effectiveness against laboratory-confirmed influenza hospitalizations among young children during the 2010-11 to 2013-14 influenza seasons in Ontario, Canada
Source: PLoS One. 2017 Nov 17;12(11):e0187834. doi: 10.1371/journal.pone.0187834 (PMC5693284; doi:10.1371/journal.pone.0187834)
Supplement: S5 Table — (DOCX) [file pone.0187834.s005.docx]

**S5 Table.** Additional characteristics of fully vaccinated, partially vaccinated, and unvaccinated hospitalized children

| **Characteristic** | **Fully vaccinated (n=595)** | **Partially vaccinated (n=556)** | **Unvaccinated (n=8,831)** | **p-value** |
| --- | --- | --- | --- | --- |
| Complex chronic conditions |  |  |  |  |
| Neurologic/Neuromuscular | 45 (7.6%) | 42 (7.6%) | 389 (4.4%) | <.001 |
| Cardiovascular | 69 (11.6%) | 43 (7.7%) | 461 (5.2%) | <.001 |
| Respiratory | 57 (9.6%) | 26 (4.7%) | 315 (3.6%) | <.001 |
| Urorenal | 27 (4.5%) | 27 (4.9%) | 221 (2.5%) | <.001 |
| Gastrointestinal | 72 (12.1%) | 43 (7.7%) | 397 (4.5%) | <.001 |
| Hematologic/Immunodeficiency | 46 (7.7%) | 31 (5.6%) | 351 (4.0%) | <.001 |
| Metabolic | 32 (5.4%) | 27 (4.9%) | 216 (2.4%) | <.001 |
| Premature/Neonatal | 108 (18.2%) | 77 (13.8%) | 821 (9.3%) | <.001 |
| Other Congenital | 176 (29.6%) | 133 (23.9%) | 1422 (16.1%) | <.001 |
| Other/Not specified | 71 (11.9%) | 44 (7.9%) | 416 (4.7%) | <.001 |
| Technology Assistance | 71 (11.9%) | 49 (8.8%) | 444 (5.0%) | <.001 |
|  |  |  |  |  |
| ICU admission | 52 (8.7%) | 44 (7.9%) | 531 (6.0%) | 0.008 |
|  |  |  |  |  |
| Past ICU admission | 68 (11.4%) | 95 (17.1%) | 977 (11.1%) | <.001 |

ICU, intensive care unit
