# Supplementary material for: Vaccine effectiveness against laboratory-confirmed influenza hospitalizations among young children during the 2010-11 to 2013-14 influenza seasons in Ontario, Canada
Source: PLoS One. 2017 Nov 17;12(11):e0187834. doi: 10.1371/journal.pone.0187834 (PMC5693284; doi:10.1371/journal.pone.0187834)
Supplement: S6 Table — (DOCX) [file pone.0187834.s006.docx]

**S6 Table.** Unadjusted vaccine effectiveness estimates

| **Analysis (cases/total)** | **Fully vaccinated** | **Partially vaccinated** | **Any vaccination** |
| --- | --- | --- | --- |
| Overall (1280/9982) | 59.0 (42.3, 70.9) | 43.9 (23.5, 58.9) | 51.8 (39.1, 61.9) |
| *Season* |  |  |  |
| 2010-11 (369/2537) | 72.0 (35.9, 87.8) | 69.4 (34.0, 85.8) | 70.7 (48.2, 83.4) |
| 2011-12 (223/1553) | 52.7 (1.2, 77.4) | 51.4 (−6.8, 77.9) | 52.1 (17.1, 72.3) |
| 2012-13 (359/3040) | 33.8 (−15.8, 62.1) | −2.3 (−69.7, 38.4) | 17.4 (−21.2, 43.7) |
| 2013-14 (329/2852) | 71.2 (41.0, 85.9) | 49.0 (9.3, 71.3) | 60.6 (37.8, 75.0) |
| *By influenza type/subtype* |  |  |  |
| Influenza A (882/9584) | 63.9 (44.4, 76.6) | 51.6 (28.2, 67.3) | 58.0 (43.6, 68.8) |
| A/H1N1 (164/8866) | 82.3 (28.3, 95.6) | 22.4 (-58.5, 62.1) | 53.7 (11.9, 75.6) |
| A/H3N2 (287/8989) | 60.1 (19.1, 80.4) | 72.7 (33.7, 88.8) | 66.2 (40.8, 80.7) |
| Influenza B (402/9104)^a^ | 44.5 (6.3, 67.1) | 27.1 (-17.9, 55.0) | 36.2 (8.4, 55.6) |
| *By age group* |  |  |  |
| 6-23 months (618/6039) | 48.8 (14.6, 69.3) | 24.7 (−8.6, 47.8) | 34.7 (11.6, 51.8) |
| 24-59 months (662/3943) | 69.5 (51.6, 80.7) | 61.9 (30.8, 79.0) | 67.0 (52.3, 77.2) |
| *By sex* |  |  |  |
| Females (526/4257) | 63.9 (36.5, 79.5) | 55.6 (24.5, 73.9) | 59.9 (40.6, 72.9) |
| Males (754/5725) | 55.6 (31.7, 71.1) | 35.4 (5.2, 56.0) | 46.0 (27.8, 59.7) |
| *By complex chronic condition* |  |  |  |
| Yes (344/2589) | 62.1 (35.2, 77.8) | 47.0 (10.3, 68.6) | 55.5 (34.7, 69.7) |
| No (936/7393) | 58.0 (34.3, 73.1) | 43.2 (16.4, 61.4) | 50.5 (33.5, 63.2) |
| *By asthma* |  |  |  |
| Yes (392/3672) | 53.4 (20.6, 72.6) | 31.0 (−13.1, 57.9) | 43.4 (18.0, 60.9) |
| No (888/6310) | 60.7 (38.5, 74.9) | 49.5 (24.6, 66.1) | 55.1 (39.2, 66.8) |
| *Peak month* |  |  |  |
| Yes (413/2245) | 63.5 (24.2, 82.4) | 51.2 (14.2, 72.3) | 56.5 (31.6, 72.4) |
| No (867/7737) | 55.5 (34.4, 69.8) | 41.2 (14.6, 59.5) | 49.0 (32.9, 61.2) |
|  |  |  |  |
| *Sensitivity analyses* |  |  |  |
| Restricted to ARI-coded hospitalizations (1183/8760) | 57.6 (39.6, 70.2) | 40.0 (17.4, 56.5) | 49.3 (35.5, 60.2) |
|  |  |  |  |
| Included term for any comorbidity in model (1280/9982) | 57.6 (40.3, 69.9) | 42.8 (22.0, 58.1) | 50.5 (37.4, 60.9) |
|  |  |  |  |
| Other respiratory virus positive (1600/3927)^b^ | 26.6 (4.4, 43.6) | 6.3 (-22.1, 28.1) | 17.3 (-0.3, 31.8) |

^a^n=24 specimens were not tested for influenza B

^b^Restricted to those who were tested for respiratory syncytial virus, parainfluenza virus, adenovirus, and human metapneumovirus.
