# Supplementary material for: Vaccine effectiveness against laboratory-confirmed influenza hospitalizations among young children during the 2010-11 to 2013-14 influenza seasons in Ontario, Canada
Source: PLoS One. 2017 Nov 17;12(11):e0187834. doi: 10.1371/journal.pone.0187834 (PMC5693284; doi:10.1371/journal.pone.0187834)
Supplement: S1 Fig — (DOCX) [file pone.0187834.s007.docx]

**S1 Figure.** Impact of misclassification of vaccination status on VE estimates, by a) changing sensitivity and b) changing specificity

1. Impact of misclassification of vaccination status by changing sensitivity, holding specificity=98%
2. Impact of misclassification of vaccination status by changing specificity, holding sensitivity=77%

Black dot indicates VE under most likely misclassification conditions.
